# Primary Gingival Anaplastic Lymphoma Kinase (ALK)-Positive Anaplastic Large Cell Lymphoma in a Young Boy

**DOI:** 10.7759/cureus.89217

**Published:** 2025-08-01

**Authors:** Claire M Lutz, Julie Bruneau, Thierry J Molina, Anne-Laure Ejeil

**Affiliations:** 1 Department of Odontology, Oral Medicine and Oral Surgery, Bretonneau Hospital, Assistance Publique-Hôpitaux de Paris (AP-HP) Université Paris Cité, Paris, FRA; 2 Department of Pathology, Robert Debré and Necker-Enfants Malades University Hospitals, Assistance Publique-Hôpitaux de Paris (AP‑HP) Université Paris Cité, Paris, FRA

**Keywords:** anaplastic large cell lymphoma, head and neck cancer, oral cancer, pediatric lymphoma, primary oral lymphoma

## Abstract

Anaplastic large cell lymphoma (ALCL) is a rare non-Hodgkin lymphoma derived from natural killer (NK) or T-cells. It mainly affects lymph nodes, and less frequently, extranodal sites such as the skin, bone, or oral cavity. Lack of awareness of these extranodal manifestations can lead to delayed diagnosis, sometimes with dramatic consequences. Here, we report the case of a young boy referred to our oral pathology consultation with persistent intraoral and cervical swellings that had lasted for three months, despite multiple dental and medical appointments and treatments. He was previously misdiagnosed repeatedly with infectious cellulitis of dental origin. Considering the clinical features, we suspected a malignant process and referred the patient to a pediatric oncohematology department. Anatomopathological analyses revealed an anaplastic lymphoma kinase (ALK)-positive ALCL, and appropriate treatment could be initiated. Extranodal manifestations, particularly oral, are little-known forms of lymphoma, especially in children. In most cases of extranodal ALCL, the first diagnosis considered is a benign tumor, which delays the correct diagnosis. It is therefore essential to raise awareness among practitioners about the detection of extranodal, particularly oral, manifestations of these rare lymphomas.

## Introduction

Lymphomas are a heterogeneous group of lymphoid malignancies broadly classified into Hodgkin and non‑Hodgkin lymphomas (NHL), with NHL accounting for about 90% of all lymphomas. NHLs include B‑cell lymphomas, which represent the vast majority, and T‑cell lymphomas, which are less frequent (10-15%). Anaplastic large-cell lymphoma (ALCL), a rare T-cell NHL, represents less than 10% of all NHL and about 12% of T-cell lymphomas, most commonly affecting children and young adults [1, 2]. There are two types: cutaneous ALCL and systemic ALCL. The latter includes two entities according to the expression of anaplastic lymphoma kinase (ALK): the ALK-positive and ALK-negative ALCL. These T-cell lymphomas are characterized histologically by the proliferation of CD30-positive large lymphoid cells. Their diagnosis is often delayed, complicating patient care, particularly because they are considered aggressive tumors [1, 2]. The main symptoms of this malignancy are chronic lymphadenopathy, severe fatigue, night sweats, fever, and weight loss. Interestingly, primary extranodal manifestations, especially those affecting the oral mucosa, are associated with even longer delays in diagnosis [3, 4]. Primary ALCL of the oral cavity is rare, with only a few cases reported in the literature. Moreover, the oral manifestations of ALCL can mimic other benign oral pathologies.

Herein, we report the case of a young boy whose initial clinical manifestation of ALK‑positive ALCL was located in the gingiva, highlighting the diagnostic challenges associated with such an uncommon oral presentation.

## Case presentation

A child aged seven years and nine months was referred to our oral pathology consultation by his pediatric dentist for a left-sided cervical swelling associated with an ipsilateral ulcerated gingival mass in the mandible, present for the past three months. This presentation followed a complex and ultimately inconclusive diagnostic history. The patient’s initial dental consultation focused on the resorption of the deciduous lower left canine, attributed to an oral lesion that had persisted for several weeks. At that time, the dentist prescribed antibiotics and performed an avulsion of the deciduous lower left canine one week later, presuming infectious cellulitis. However, the patient’s condition worsened over the following three weeks, with increasing left mandibular subangular swelling. Despite the dentist’s confirmation of cellulitis and continued antibiotic therapy for several weeks, the oral and submandibular swelling persisted. Seeking further management, the patient went to the ED of a hospital on day 34 after the initial dental consultation, still experiencing persistent swelling and delayed healing around the extraction site. Over the next month, this pattern of presumed cellulitis, antibiotic prescription, and lack of improvement was repeated at two other hospitals. While one hospital briefly considered an alternative unknown diagnosis and prescribed a cervical ultrasound, no specific follow-up appointment was scheduled. Finally, in light of the persistent gingival mass and cervical swelling, he was referred to our consultation.

The patient’s medical history was unremarkable. The anamnesis indicated painful limitation in extending the left arm, which appeared concurrently with the oral mass. He also exhibited feeding difficulties due to dysphagia, although there was no deterioration in his general condition or weight loss. There had been a single episode of loss of consciousness three weeks earlier, which occurred without any identifiable trigger or prodromal symptoms.

**Figure 1:**
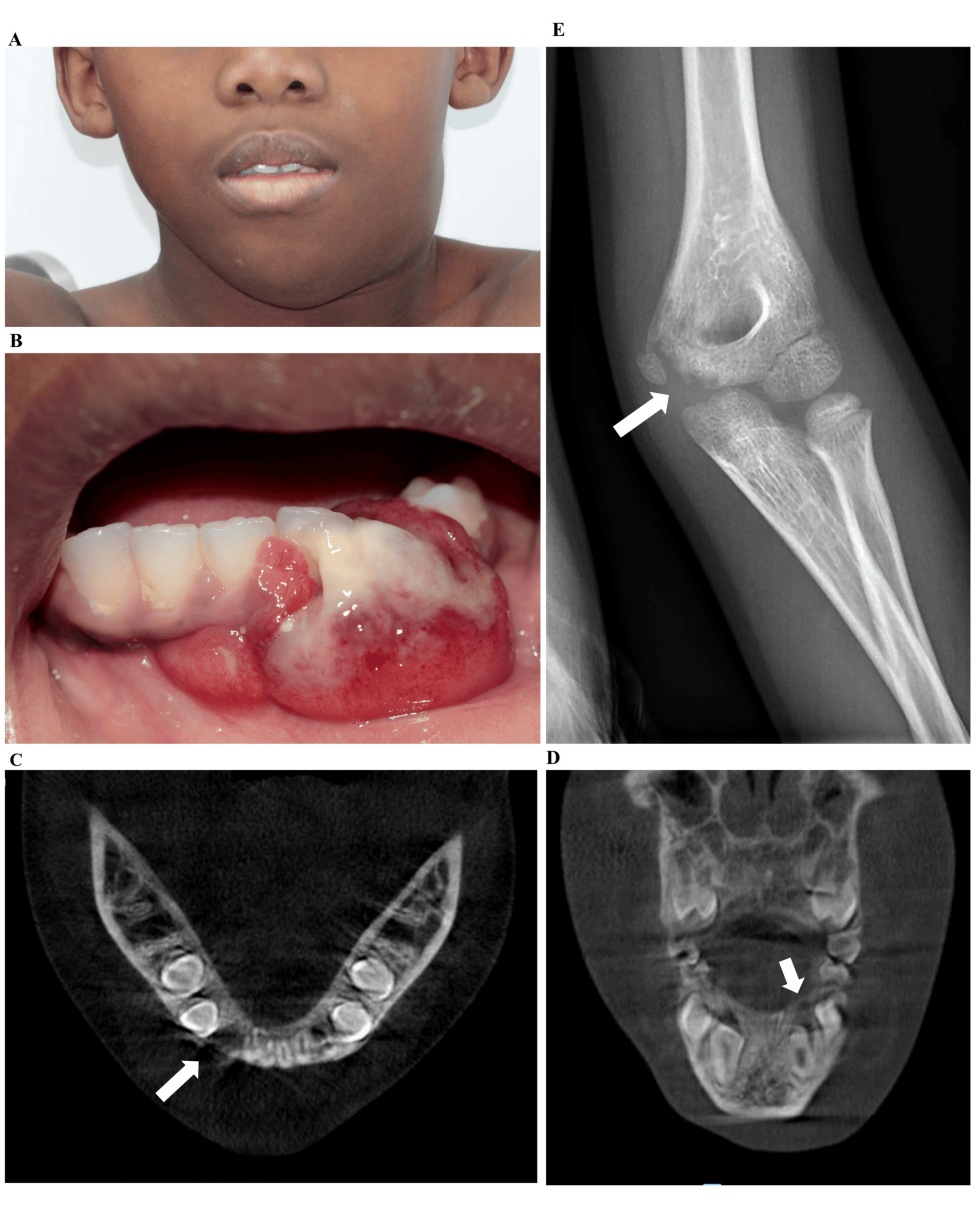
Clinical and radiographic features of primary mandibular ALK-positive anaplastic large cell lymphoma. (A) Extraoral examination showing a left cervico-mandibular swelling. (B) Intraoral examination showing a reddish, ulcerated, necrotic mass on the lower left gingiva and alveolar ridge. (C) Cone Beam Computed Tomography (CBCT), axial section, and (D) coronal section showing discrete bone resorption of the alveolar bone (arrows). (E) Radiograph of the left arm showing osteolysis of the cortical bone of the humeral epiphysis (arrow). ALK: Anaplastic lymphoma kinase.

Extraoral examination revealed a large, firm, painful, and fixed left-sided mandibular swelling, extending from the angle to the chin, causing restricted cervical rotation and limiting mouth opening to 3 cm (Figure 1A). Intraoral examination revealed a reddish, ulcerated, necrotic mass on the lower gingiva, adjacent to the socket of the deciduous canine extracted two months prior (Figure 1B). Both the buccal swelling and the associated lingual floor were firm and painful on palpation. Cone Beam Computed Tomography taken in our department showed discrete and superficial bone resorption of the mandible (Figures 1C-1D). An X-ray of the left arm, prescribed due to the painful limitation, revealed lysis of the humeral epiphysis cortical bone (Figure 1E), while the cervical ultrasound prescribed ten days earlier by a pediatric department showed two left submandibular lymphadenopathies (27 × 17 mm and 11 × 20 mm). Routine laboratory studies did not show any abnormalities.

When faced with a non-resolving gingival mass, several diagnoses should be considered. In children, a pyogenic granuloma following extraction of the deciduous canine may occur. An epulis, a peripheral giant cell granuloma, or an ossifying fibroma are also possible. However, the clinical presentation of an ulcerative mass, combined with fixed, firm adenopathies on palpation and bone lesions, strongly suggested a malignant hematologic process. Therefore, the child was urgently referred to the oncohematology department of a children's hospital for specialized care. A biopsy of the mandibular mass was performed by the oncohematology department to confirm hematologic malignancy.

**Figure 2:**
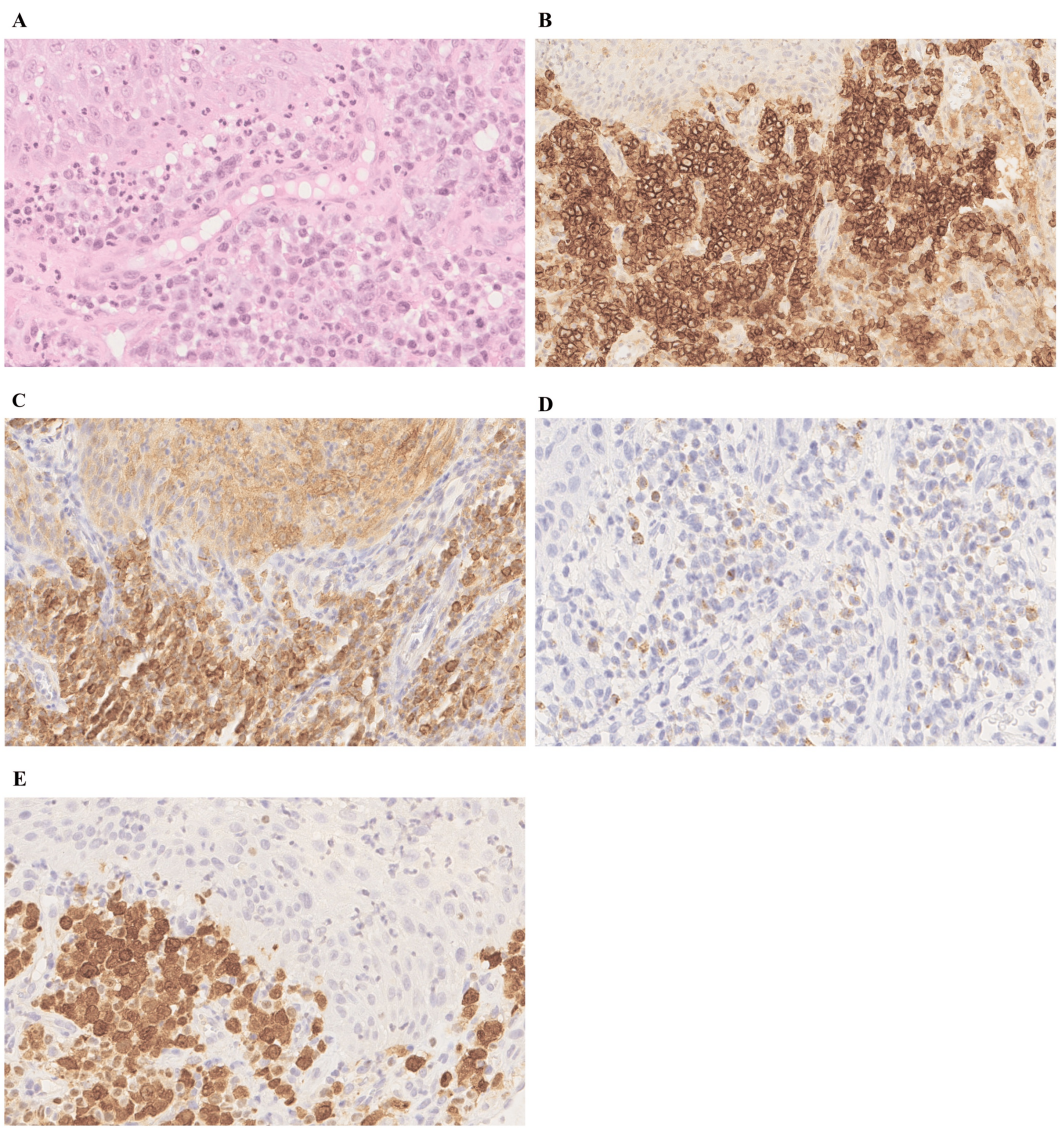
Histopathological features of primary mandibular ALK-positive anaplastic large cell lymphoma. (A) Tumor cells arranged in sheets are present in the lamina propria (lower right) beneath normal Malpighian epithelial cells of the oral mucosa (upper left) (H&E stain, G × 400). (B) Tumor cells express CD30 (anti-CD30, G × 200). (C) Tumor cells show ALK expression with a nuclear and cytoplasmic pattern (anti-ALK, G × 400). (D) Tumor cells express EMA (anti-EMA, G × 400). (E) Granzyme B expression in tumor cells (anti-Granzyme B, G × 400). ALK: Anaplastic lymphoma kinase.

Histopathological examination exhibited an infiltrate of medium to large lymphoid cells with eosinophilic cytoplasm and reniform nuclei (Figure 2A). Immunohistochemistry showed that these lymphoid cells expressed CD30 (a hallmark feature of ALCL) (Figure 2B), ALK in a nuclear and cytoplasmic pattern (suggesting that the translocation partner responsible for ALK overexpression is nucleophosmin [NPM1]) (Figure 2C), EMA (epithelial membrane antigen, almost always positive in ALK+ ALCL) (Figure 2D), and Granzyme B (partially, supporting the active cytotoxic phenotype of the tumor cells) (Figure 2E). They were negative for CD3 and CD5 (T-cell markers) and CD20 (B-cell marker). The Ki67 proliferation index was 70%. Additional staging procedures performed in the pediatric oncohematology department revealed cervical, cutaneous, digestive, bone, and pulmonary involvement. The bone marrow was intact, but a positive minimal disseminated disease (MDD) was observed.

Based on clinical, MRI, and histopathological findings (conducted in the oncohematology department), a diagnosis of stage IV primary ALK-positive anaplastic large cell T/NK lymphoma of the mandible was established.

Treatment

**Figure 3:**
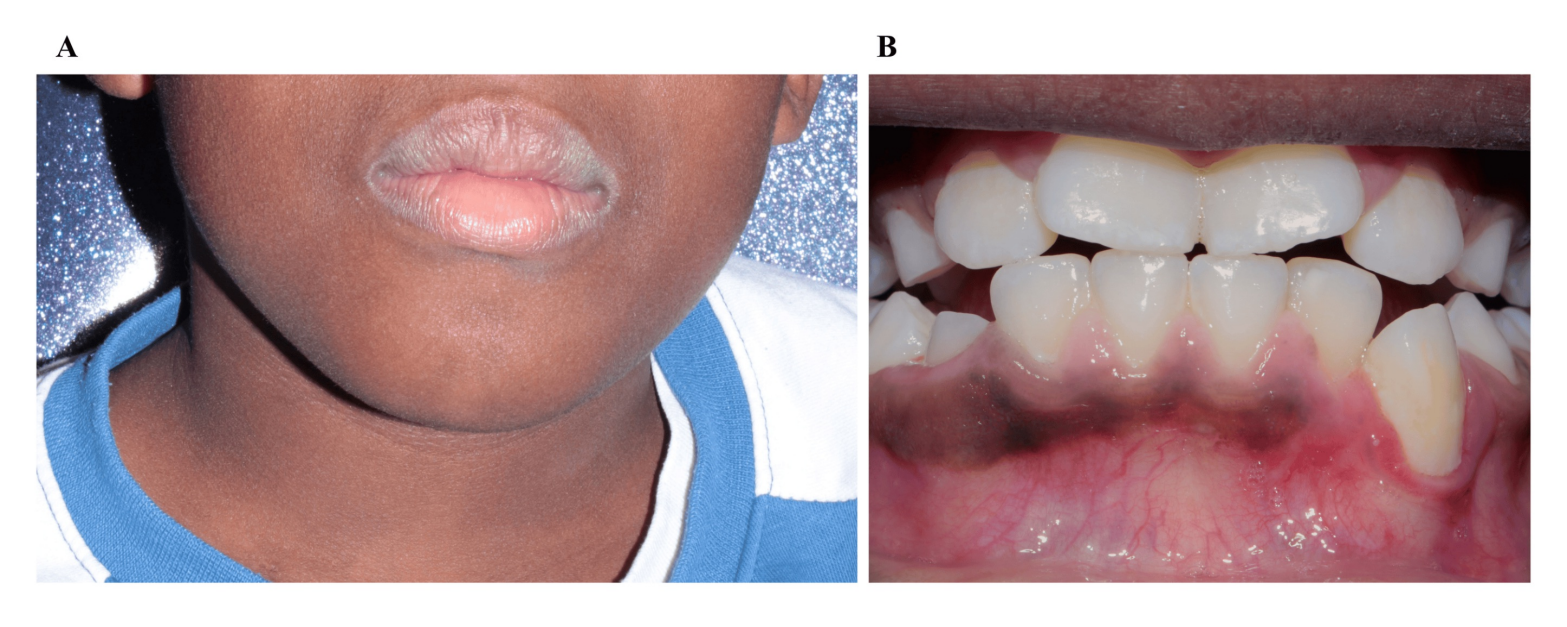
Clinical appearance of the patient after one year of treatment for primary mandibular ALK-positive anaplastic large cell lymphoma. (A) Extraoral examination showing regression of the left cervico-mandibular swelling. (B) Intraoral examination showing regression of the lower left gingival mass. ALK: Anaplastic lymphoma kinase.

A first-line treatment of polychemotherapy, according to the ALCL 99 protocol (Course P: dexamethasone, endoxan, methotrexate, and aracytine; Course AM1: dexamethasone, ifosfamide, methotrexate, cytarabine, etoposide; Course BM1: dexamethasone, methotrexate, cyclophosphamide, doxorubicin) was initiated [5]. Tolerance was rather poor, with nausea/vomiting, abdominal pain, and asthenia. The first courses of chemotherapy led to a good clinical response and a partial morphological response on the cervico-thoraco-abdomino-pelvic CT and mandibular MRI. However, given the persistence of a positive minimal residual disease (MRD, 0.14%) at two months, a sign of poor disease control, long-term targeted therapy with an ALK inhibitor (alectinib 150 mg twice daily for seven days, followed by 150 mg in the morning and 300 mg in the evening) was initiated. This led to the resolution of MRD after one month of treatment, with good compliance and no specific adverse events. The patient also showed regression of the cervical masses and the gingival nodule (Figures 3A-3B). Unfortunately, two months after discontinuation of treatment following two years of remission, the patient experienced a subdiaphragmatic relapse. Reintroduction of alectinib led to a new complete remission ten months after relapse, which is still ongoing at the time of writing.

## Discussion

ALCL is a subgroup of NK/T-cell NHL, first described by Stein H et al. [6] in 1985, accounting for less than 10% of NHL. Two systemic subtypes have been recognized: ALK-positive ALCL and ALK-negative ALCL.

ALK-positive ALCL commonly affects male patients in their first decades of life, frequently during childhood, and often presents with peripheral lymph node involvement and B symptoms (fever, night sweats, asthenia, and weight loss) [1,2]. In contrast, ALK-negative ALCL typically affects older patients [1,3,4]. The higher incidence of ALK-positive ALCL in children is attributed to a chromosomal translocation involving the NPM1-ALK oncogene [7]. Extranodal involvement, which can occur with or without lymph node involvement, is known in NHLs, including ALCL, and is often associated with a high grade at diagnosis [3,4], as observed in our patient who presented with a primary oral manifestation. Primary oral involvement in ALCL is extremely rare, accounting for less than 1% of cases, with approximately thirty cases reported in the English literature. In systemic ALK-positive ALCL, extranodal involvement is most commonly cutaneous, pulmonary, digestive, hepatic, or osseous [8]. In this case, the patient’s elbow pain was found to be due to secondary bone involvement of ALK-positive ALCL, with further examination revealing pulmonary, digestive, and skin involvement.

While ALK-positive intraoral involvement is less frequent than ALK-negative, both represent rare localizations of ALCL. They are mainly located on the gingiva/alveolar ridge or the palate. Reflecting the locally aggressive behavior often observed in oral ALCL, our patient presented with an ulcerated, painful nodule associated with underlying bone resorption, consistent with findings reported in the literature [4] and similar to the case of an 18-year-old male with ALK-positive ALCL described by de Andrade BA et al. [8]. However, this case highlighted an unusual manifestation of oral ALK-positive ALCL associated with arm pain and humeral lysis. The diagnostic delay, attributed to iterative misdiagnoses of odontogenic cellulitis, was approximately eight weeks in our case, comparable to the 60-day median time to diagnosis reported in pediatric cohorts [2]. In our case, routine blood tests were unremarkable, while early imaging revealed bone involvement. This illustrates that routine laboratory work‑up may appear normal despite aggressive underlying disease, contributing to diagnostic delay, as reported in other oral ALCL cases [3,4].

Diagnosing ALCL involves a multi-faceted approach. Initially, a biopsy of sufficient size (>5 mm) is crucial, followed by a thorough evaluation for MDD through blood tests, bone marrow aspirates, and lumbar puncture. Microscopic analysis shows a proliferation of large atypical lymphoid cells with abundant cytoplasm and eccentric horseshoe- or kidney-shaped nuclei, a pattern observed in our patient. The characterization of these cells involves immunohistochemical markers, with strong expression of CD30. ALK expression is observed in about 50% of systemic ALCL and is the result of a t(2;5) (NPM1/ALK) chromosomal translocation in the majority of cases. In contrast, expression of other markers such as CD2, CD5, CD3, CD45, or cytotoxic markers such as TIA1, Granzyme B, and perforin is more variable [2]. This lack of uniform marker expression contributes to the diagnostic challenges associated with ALCL, as it can be easily confused with poorly differentiated squamous cell carcinoma, melanoma, Hodgkin lymphoma, or anaplastic diffuse large B-cell lymphoma [1,8].

Treatment includes a combination of chemotherapies, leading to excellent outcomes, with 5‑year overall survival exceeding 90% and event‑free survival around 74% under current protocols such as ALCL 99 [5]. However, relapses still occur in about 30% of patients, usually within the first two years. These chemotherapies are also extremely aggressive and associated with major toxicities. During treatment, our patient experienced significant discomfort, including nausea, vomiting, abdominal pain, and asthenia, highlighting the common but challenging side effects of chemotherapy. Recently, targeted therapies have emerged, notably anti-ALK drugs such as alectinib. These can be administered long-term or transiently before allogeneic hematopoietic stem cell transplantation [2]. Such treatments achieve durable control of the disease with fewer adverse effects but remain second-line treatments (for children with relapsed ALCL or as consolidation therapy). Following second-line treatment with the ALK inhibitor alectinib, introduced for persistent MRD, our patient achieved a rapid and well‑tolerated complete remission maintained for two years. Two months after discontinuation of treatment, he relapsed, but reinitiation of alectinib led to a second complete remission within ten months, which is still ongoing.

ALK-positive ALCL is a distinct entity with a better prognosis than ALK-negative ALCL [4,8]. Young age, chemotherapy treatment, and extra-nodal head and neck primary site involvement are known to be associated with more favorable prognosis [3]. Our patient required second-line treatment, likely due to the advanced stage of the disease.

The rarity of ALCL and the lack of awareness about its oral manifestations may explain the delayed diagnosis and advanced disease stage at presentation. This delay is concerning given the potentially aggressive nature of the pathology and the increased risk of treatment failure. The median time to diagnosis is approximately sixty days, which can be even longer in pediatric cases, leading to potentially greater harm and long-term consequences [9]. By including ALCL in the differential diagnosis, particularly in cases with persistent symptoms, pediatric dentists can help facilitate earlier diagnosis and intervention, potentially improving outcomes for affected children.

This delay is partly attributable to the clinical presentation mimicking benign lesions or locoregional infections, as reported by other authors [4,10]. In many of the cases described in the literature, the proposed differential diagnoses were entirely benign, such as pyogenic granuloma, peripheral giant cell granuloma, peripheral ossifying fibroma, or fungal/bacterial infections [4,10,11], based solely on clinical evaluation. Malignancy is generally diagnosed only after the failure of first-line treatment or incidentally discovered during biopsy, usually performed in specialized oral medicine or hemato-oncology hospital departments. Although histological analysis (e.g., immunohistochemistry) is costly, it remains essential for diagnosing malignancy. Given the persistent and worsening symptoms, a malignancy and biopsy should have been considered earlier in our case.

While extraoral manifestations can raise suspicion of malignancy, routine blood tests (e.g., CBC and CRP) often offer limited diagnostic value, as they frequently show no abnormalities [4,8,10,12] or only non-specific findings indicative of an inflammatory syndrome [13,14]. In our patient’s case, blood tests were normal despite the aggressive disease progression. Therefore, collaboration between physicians, oral surgeons, and pediatric dentists is essential to accelerate the diagnosis and treatment of these patients, especially when the initial therapy, guided by a presumed benign diagnosis, fails to produce improvement.

## Conclusions

In summary, we presented a rare pediatric case of ALCL in the oral cavity, initially misdiagnosed as infectious cellulitis. This highlights a critical challenge: oral ALCL often mimics benign pathologies, underscoring the importance of biopsy for definitive diagnosis. Pediatric dentists and oral surgeons play a pivotal role in early recognition. By including ALCL in the differential diagnosis, particularly in cases of persistent or atypical oral lesions, they can facilitate timely intervention and potentially improve outcomes for affected children. This case emphasizes the need for a structured, proactive diagnostic approach, including prompt referral to specialists when first-line treatments fail. Collaborative efforts between physicians and oral health professionals are essential to ensure the timely diagnosis and effective management of this rare but aggressive disease.
